# Adherence to Antiretroviral Therapy and Its Effect on Survival of HIV-Infected Individuals in Jharkhand, India

**DOI:** 10.1371/journal.pone.0066860

**Published:** 2013-06-18

**Authors:** Sandeep Rai, Bidhubhusan Mahapatra, Subhashish Sircar, Pinnamaneni Yujwal Raj, Srinivasan Venkatesh, Mohammed Shaukat, Bharat Bhusan Rewari

**Affiliations:** 1 Jharkhand and Bihar State AIDS Control Society, Ranchi, India; 2 HIV and AIDS Program, Population Council, New Delhi, India; 3 National AIDS Control Organization, Department of AIDS Control, Ministry of Health and Family Welfare, Government of India, New Delhi, India; University of Illinois at Chicago, United States of America

## Abstract

**Introduction:**

Research in India has extensively examined the factors associated with non-adherence to antiretroviral therapy (ART) with limited focus on examining the relationship between adherence to ART regimen and survival status of HIV infected patients. This study examines the effect of optimal adherence to ART on survival status of HIV infected patients attending ART centers in Jharkhand, India.

**Materials and Methods:**

Data from a cohort of 239 HIV infected individuals who were initiated ART in 2007 were compiled from medical records retrospectively for 36 months. Socio-demographic characteristics, CD4 T cell count, presence of opportunistic infections at the time of ART initiation and ART regimen intake and survival status was collected periodically. Optimal adherence was assessed using pill count methods; patients who took <95% of the specified regimens were identified as non-adherent. Cox-proportional hazard model was used to determine the relative hazards of mortality.

**Results:**

More than three-fourths of the patients were male, on an average 34 year old and median CD4 T cell count was 118 cells/cmm at the time of ART registration. About 57% of the patients registered for ART were found to be adherent to ART. A total of 104 patients died in 358.5 patient-years of observation resulting in a mortality rate of 29 per 100 patient-years (95% confidence interval (CI): 23.9–35.2) and median survival time of 6.5 months (CI: 2.7–10.9). The mortality rate was higher among patients who were non-adherent to ART (64.5, CI: 50.5–82.4) than who were adherent (15.4, CI: 11.3–21.0). The risk of mortality was fourfold higher among individuals who were non-adherent to ART than who were adherent (Adjusted hazard ratio: 3.9, CI: 2.6–6.0).

**Conclusion:**

Adherence to ART is associated with a higher chance of survival of HIV infected patients, ascertaining the need for interventions to improve the ART adherence and early initiation of ART.

## Introduction

In the absence of a cure for HIV, the introduction of antiretroviral therapy (ART) has been able to improve the survival of HIV infected individuals [1]–[3] by delaying the progression to AIDS [4], [5]. However, the success of ART in extending the survival period of an individual depends on whether the individual adhere to the process of medication as per the prescribed treatment regimen [5]–[8]. Empirical research suggests that globally around 33% to 38% of HIV infected adults do not adhere to treatment regimens of ART [9]. Non-adherence rates to ART regimen in India varies quite considerably from 14%–86% [10]–[15].

The ART program in India was launched in 2004 and free services are being provided across 230 ART centers across the country [10]. The eligibility criteria for ART entry are: world health organization (WHO) clinical stage IV irrespective of CD4 T cell count or CD4 T cell count <200 cells/cmm or WHO clinical stage III and CD4 T cell count 200–350 cells/cmm [10]. Patients are given drugs for 30 days and asked to return to the health center after 4 weeks for a follow up evaluation and to collect drugs for the next 30 days. The generic fixed drug combination of zidovudine (or) stavudine, lamivudine and nevirapine (or) efavirenz was provided as first-line ART regimen [10], [16].

Extensive research has been undertaken in India to understand the underlying factors associated with non-adherence to ART [7]. Few studies have also examined the effect of CD4 T cell count on survival status of individuals who were on ART [10]. However, there is a dearth of evidence-based research in India in understanding the relationship between adherence to ART regimen and survival status of HIV infected patients. This study is based in the state of Jharkhand; located in the eastern part of India and largely dominated by tribal population. The estimated adult HIV prevalence was 0.13% (male: 0.16%, female: 0.10%) in 2009 [17] and about 7000 people are living with HIV. There are six ART centers in the state catering to about 3000 HIV positive individual eligible for ART.[17]. Accessibility to health services is a major challenge in the state due to poor transportation facility. Moreover, the low literacy level and poor knowledge about HIV and HIV services may make it difficult for HIV infected individuals to access services in appropriate time [18]. A large scale household survey suggested that only 17% and 25% currently married women have heard about STIs and HIV, respectively [18]. Moreover, empirical research in India have reported conflicting trends in mortality rates; few studies reported increasing mortality trends among HIV infected individuals while another set of studies reported a decline in mortality rates over the years [19]–[22]. This has prompted researchers and policy makers to questions on contributions of ART on survival status of HIV infected individuals [22]. This study, therefore, examines the correlates of survival status of HIV infected individuals who were on ART treatment, with special reference to optimal ART adherence.

## Materials and Methods

### Data

Data were compiled retrospectively from existing medical records at the ART center located in Ranchi, the capital city of Jharkhand state in India. The ART intake and clinical diagnosis history of each patient is stored in a clinical record form at the ART centers for the purpose of program monitoring and follow-up with patients. These records do not contain any personal identifiers like name or address to ensure their confidentiality. The number of patients registered at this ART center accounted for more than 55% of the state's total. At the time of ART registration, each HIV infected individuals were given drugs for two weeks. Those found adherent to drugs for those two weeks were supplied with drugs for four weeks and asked to return to the ART center again after four weeks for follow-up evaluation and collection of drugs for next four weeks. Standardized tools were used to record information on adherence status, CD4 T cell count, opportunistic infections (OIs), adverse effects and laboratory results at each visit. More details on the process of data gathering can be found elsewhere [10]. Data collected using paper formats were converted to an electronic format, entered using MS Excel and later on converted to STATA for statistical analyses. All HIV infected individuals who were 18 years or older and registered for ART during the year 2007 and whose records were readily available were considered for this study.

**Figure 1:**
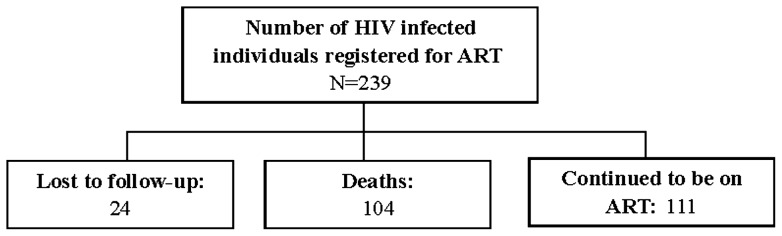
Flow chart depicting status of HIV infected individuals who were put on ART at the end of study period.

A total of 239 individuals were registered for ART during January to December 2007 (Figure 1). After 36 months of follow-up of this cohort, 104 deaths occurred to individuals, 24 lost to follow-up and remaining 111 individuals remained on ART. An individual who did not turn up at ART center for three consecutive months were considered as lost to follow-up. Because this was an observational study, a priori statistical power and sample size calculations were not conducted.

### Ethics statement

The Jharkhand state AIDS control society (JSACS) under the guidance of National AIDS Control Organization (NACO) has provided general oversight and approval for the collection and use of routine program data for examining the factors associated with survival status of ART patients. Patients are informed (as per the guidelines of national ART program in India) at the time of enrollment to ART centers that their medical history may be used for program strengthening. Verbal consent was taken from each patient at the time of enrollment who had agreed to use of their medical history for research purpose. All the patients were explained about the harms and benefits of being enrolled into ART and also their schedule of visits to ART centers. The counselor at the ART center recorded all these procedures. As the data presented in the study are for the purpose of program monitoring and program beneficiaries were informed about this apriori; therefore, the study was determined to be exempted for review from an institutional review board.

### Measures

#### Adherence status

Adherence to ART was assessed using the pill count approach. The unused pills were noted down for each patient on their subsequent visit. The number of unused pills for the entire period of follow-up was obtained by totaling the pills unused in each month (cumulative unused pills). Similar to the approach used for measurement of adherence in other studies [16], we also defined percentage drug adherence as the total number of follow-up months multiplied by 60 pills minus the cumulative number of unused pills for the entire follow-up period multiplied by 100. Patients suffering from concomitant tuberculosis (TB) were required to take additional pills and hence, appropriate adjustment was done in pill count while deciding the adherence status. We defined optimal adherence to ART if percentage drug adherence was more than 95% (coded as 1); else considered as non-adherent (coded as 0). Empirical research has recognized 95% drug regimen intake as optimum level of adherence required for antiretrovirals to work effectively against the disease [23].

#### Survival status

For each patient, information on their treatment status was recorded at the end of every month. The options for treatment status included: ‘alive and on treatment’, ‘missed picking up drugs this month’, ‘stopped temporarily’, ‘lost to follow-up’ (if patients missed visits for 3 months consecutively) and ‘died’. These response options were grouped into three categories: died, alive and on treatment and lost to follow-up which includes options: missed picking up drugs this month, stopped temporarily and lost to follow-up.

#### Socio-demographic and health-related covariates

At the time of ART registration, information on age (grouped into two categories: <35 years, 35+years); sex (male, female); education (no formal education, formal education), household income (<2000 INR, 2000+ INR (50 INR  = 1USD)); source of referral (self/hospital, voluntary counseling and treatment center/Non-governmental organization); CD4 T cell count (categorized as <200 cells/cmm, 200+ cells/cmm) were collected. Information on opportunistic infections during study period were also collected and coded as a binary variable. All these variables were included as covariates in the multivariate analyses.

### Statistical analyses

Univariate, bivariate and multivariate analyses were performed. The primary outcome of interest was survival status of individuals. Survival time was measured as duration between the date of ART initiation to the reported date of death for patients who died or the date of the last recorded visit for patients who were censored (either alive till the end of the study period or lost to follow-up). For the comparison of unadjusted survival rates, Kaplan-Meier methods were used and patients were stratified by different population sub-groups. We used the Wilcoxon log rank tests to assess statistical differences between the groups. To visually present the product limit estimates of the cumulative survival rate, Kaplan-Meier curves were generated to show survival over the study period of 36 months. Cox-proportional hazards regression models were used to investigate factors associated with survival status and results were presented in terms of hazard ratio with 95% confidence interval. The proportional hazards assumption was tested using Schoenfeld residuals. All the analyses were conducted using STATA version 11.1.

## Results

More than three-fourth (76%) of the study participants were male, on an average 35 year old and two-third (67%) of them were having formal education (Table 1). The median income of their family was 2000 INR (approximately 50 INR  = 1 USD) per month. Less than one-fifth (18%) had a CD4 T cell count of 200 cells/cmm or more at the time of ART initiation; with a median CD4 T cell count of 118 cells/cmm. Median CD4 T cell count values were higher among females (158 cells/cmm) as compared to males (109 cells/cmm) when they initiated the treatment. Furthermore, half of the patients had opportunistic infections at the time ART initiation. Overall, only about 57% of the study participants were found to be adherent to ART regimen; and adherence was higher among patients with CD4 T cell count of 200+ cells/cmm as compared to those with lesser CD4 T cell count (68% vs. 54%, P = 0.083).

**Table 1:** Profile of HIV infected individuals who were registered for ART in 2007, their median CD4 T cell count at the time of ART registration and percent with >95% mean adherence level during the study period, Jharkhand, India.

Background characteristics	Number	% or Mean (SD)	Median CD4 T cell count (cells/cmm)	Optimal adherence* (%)
**Age at ART registration (in years)**				P = 0.812
<35	117	49.0	121.0	57.3
35+	122	51.0	115.5	55.7
Mean (SD)		34.5 (7.5)		
**Sex**				P = 0.328
Male	182	76.2	108.5	58.2
Female	57	23.8	158.0	50.9
**Education**				P = 0.987
No formal education	78	32.6	142.5	56.4
Formal education	161	67.4	116.0	56.5
**Household income (in INR)**				P = 0.663
<2000	98	41.0	123.5	58.2
2000+	141	59.0	116.0	55.3
**Source of referral**				P = 0.237
Government hospital/self	91	38.1	107.0	51.6
VCTC/NGO	148	61.9	120.5	59.5
**Opportunistic infections**				P = 0.200
No	122	51.0	132.0	52.5
Yes	117	49.0	109.0	60.7
**Distance to ART center**				P = 0.140
<150 KM	118	49.4	117.5	51.7
150+ KM	121	50.6	119.0	61.2
**CD4 T cell count**				P = 0.083
<200 cells/cmm	195	81.6		53.9
200+ cells/cmm	44	18.4		68.2
**Total**	**239**	**100.0**	118.0	56.5

SD: Standard deviation

* >95% drug regimen taken

**Figure 2:**
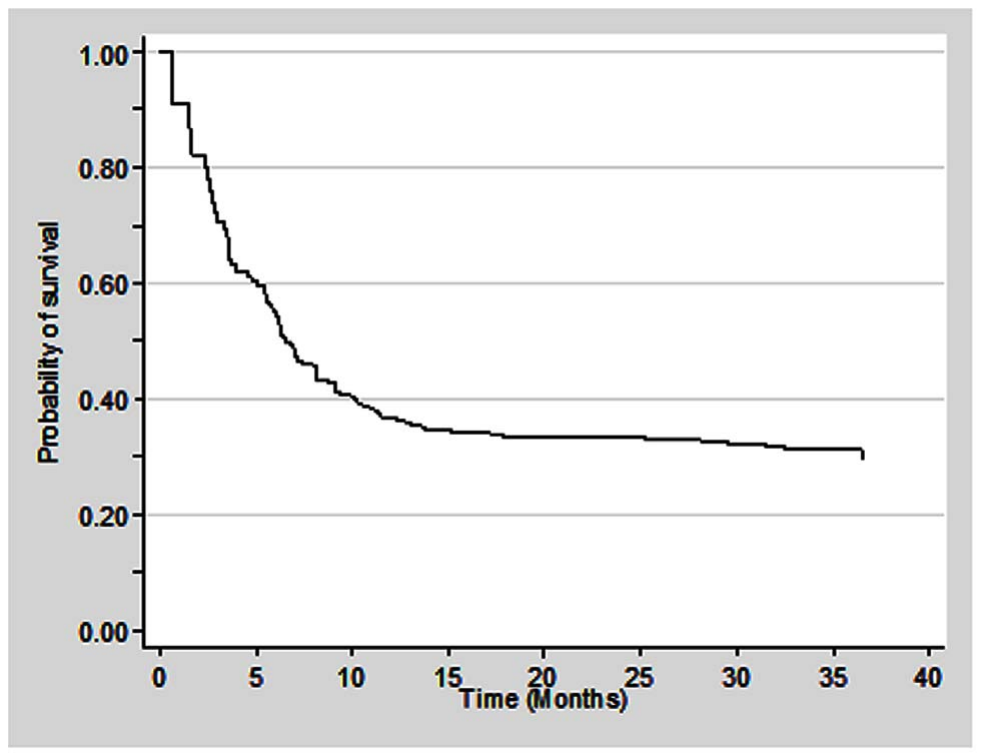
Kaplan Meier survival estimates among HIV infected individuals who were on ART (N = 239).

**Figure 3:**
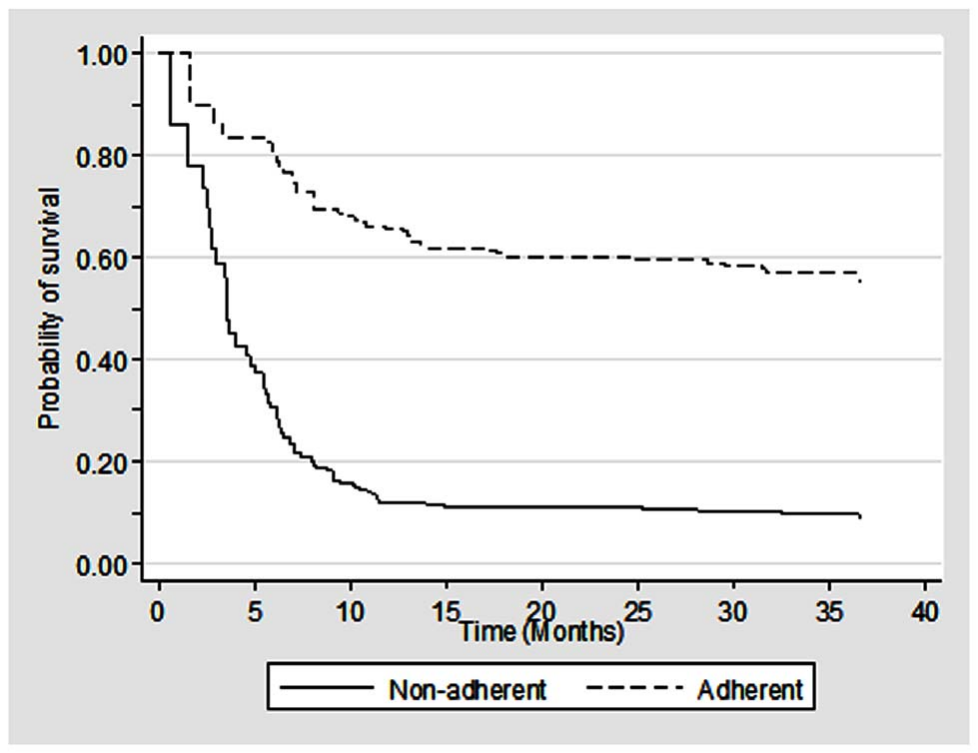
Kaplan Meier survival estimates among HIV infected individuals who were on ART by adherence status (N = 239).

**Figure 4:**
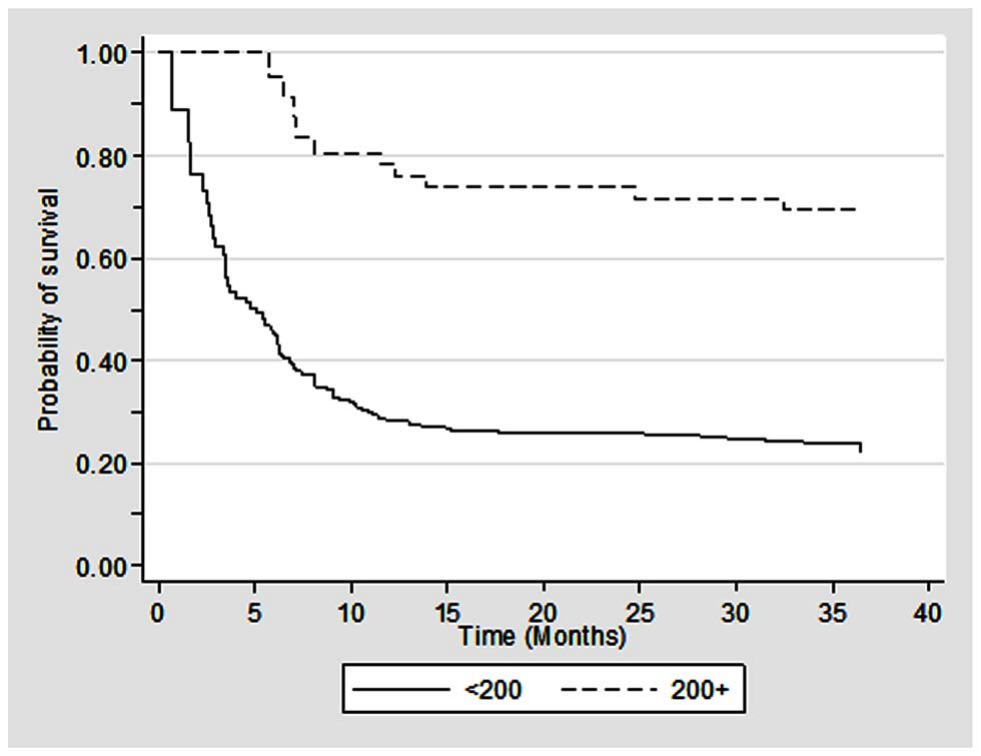
Kaplan Meier survival estimates among HIV infected individuals who were on ART by CD4 T cell count at the time of initiation of ART (N = 239).

During the study period, a total of 104 patients died in 358.5 patient-years of observation, resulting in a mortality rate of 29 per 100 patient-years (95% CI: 23.9–35.2) (Table 2). The median survival time was 6.5 months with 95% confidence interval of 2.7–10.9 (Figure 1). The survival rate at the end of 6- and 12-month was 54% and 36% respectively. The mortality rates were higher among individuals who were non-adherent to the ART regimen (64.5, 95% CI: 50.5–82.4) than who were adherent to the ART regimen (15.4, 95% CI: 11.3–21.0). Higher mortality rate was also noted among individuals who were 35+ years (39.0, 95% CI: 30.5–49.8), males (34.4, 95% CI: 27.9–42.5) and those with baseline CD4 T cell count of <200 cells/cmm (34.3, 95% CI: 28.1–42.0) than their respective counterparts. Furthermore, the Kaplan-Meier survival curves suggest significantly lower median survival time for these groups (Figures 2, 3, 4).

**Table 2:** Unadjusted all-cause mortality rates and adjusted relative hazards of all-cause mortality according to adherence categories, baseline CD4 count and other variables assessed using Cox proportional hazard model (N = 239).

Background characteristics	Number of deaths	Total patient years	Death rate per 100- patient years	Adjusted Hazard Ratio (95% CI)
**Age (in years)**				
<35	40	194.2	20.6 (15.1–28.1)	Referent
35+	64	164.3	39.0 (30.5–49.8)	1.6 (1.1–2.5)
**Sex**				
Male	86	249.9	34.4 (27.9–42.5)	2.8 (1.6–4.9)
Female	18	108.6	16.6 (10.4–26.3)	Referent
**Education**				
No formal education	36	121.9	29.5 (21.3–40.9)	Referent
Formal education	68	236.5	28.7 (22.7–36.5)	0.8 (0.5–1.2)
**Household income (in INR)**				
<2000	36	158.4	22.7 (16.4–31.5)	Referent
2000+	68	200.1	34.0 (26.8–43.1)	1.5 (0.9–2.2)
**Source of referral**				
Government hospital/self	34	138.8	24.5 (17.5–34.3)	Referent
VCTC/NGO	70	219.6	31.9 (25.2–40.3)	1.2 (0.8–1.8)
**CD4 T cell count**				
<200 cells/cmm	94	273.7	34.3 (28.1–42.0)	2.3 (1.2–4.4)
200+ cells/cmm	10	84.8	11.8 (6.3–21.9)	Referent
**Opportunistic infections**				
No	48	184.9	26.0 (19.6–34.4)	Referent
Yes	56	173.6	32.3 (24.8–41.9)	1.0 (0.6–1.4)
**Adherence status**				
<95%	64	99.2	64.5 (50.5–82.4)	3.9 (2.6–6.0)
> = 95%	40	259.3	15.4 (11.3–21.0)	Referent
**Distance to ART center**				
<150 KM	64	175.0	30.3 (23.1–39.6)	Referent
150+ KM	40	183.5	27.8 (21.1–36.6)	1.2 (0.8–1.9)
**Total**	**104**	**358.5**	**29.0 (23.9–35.2)**	

Higher survival rates were associated with young age, gender, higher CD4 T cell count and optimum adherence to ART regimen (Table 2). For example, the risk of mortality was fourfold higher among individuals who were non-adherent to the ART regimen than who were adherent to ART (Adjusted hazard ratio (adjHR): 3.9, 95% CI: 2.6–6.0). Similarly, individuals with CD4 T cell counts less than 200 cells/cmm were two times more likely to experience hazard than those with higher CD4 T cell count (adjHR: 2.3, 95% CI: 1.2–2.4).

## Discussion

This study based on 239 HIV infected patients followed-up for 36 months documented a moderate level (57%) of adherence to ART, and crude mortality rate of 29 per 100 patient-years which is higher than the adult mortality rate in general population. Furthermore, we found that the median survival time was around six months and survival rate at the end of first year of study was only 36% indicating that most of the deaths occurred during the first year of the study. The higher mortality in the first six months documented by this study corroborates the findings from several other studies [2], [10], [24], [25]. The higher mortality in the first six months could be due to poor adherence to ART regimen combined with other co-morbid infections and low CD4 T cell count. Post-hoc analysis suggests that among individuals who died in the first six months of the study, only 35% of them were adherent to ART regimen and about 50% were suffering from an opportunistic infection. In addition, the mean CD4 T cell count among these patients was 138 cells/cmm. The adherence level documented in this study was similar to the studies conducted in south India by Venkatesh et al. [12] and Cauldbeck et al. [14]; however, comparatively lower than many other studies conducted in India where rate of adherence ranged from 65%–86% [10], [11], [13], [15]. The difference in adherence level from other studies can be attributed to the way adherence was measured in those studies. For example, few of these studies used the self-report approach to assess adherence whereas others used pill count approach. The moderate level of adherence to ART is a cause of concern as it directly affects the survival status of individuals. HIV prevention program in India can adopt an approach such as in the DOTS program to control TB to improve the rate of adherence among individuals. Better counseling with follow-up by front health workers to monitor pill intake can be step forward in this direction.

The benefit of adherence to ART on prolonged survival of HIV individual is well argued and documented in other developed and developing countries [1], [2], [26], [27]. Consistent with those studies, we also noted a higher chance of survival among patients adherent to ART than who were not adherent. Non-adherent patients had a mortality of 65 deaths per 100 patient-years compared 15 deaths per 100 patient-years among adherent patients. Empirical research suggests that non-adherence to ART regimen can lead to virologic, immunologic and clinical failure and increases the risk of transmission of drug resistant virus [2], [28]. Furthermore, lower adherence to ART and higher mortality was observed among individuals with CD4 T cell count <200 cells/cmm at the time ART registration than who had a higher cell count indicating CD4 T cell count being associated with increased mortality corroborating findings from other research studies [2], [10], [24], [26], [29], [30]. In addition, about 60% of patients who were non-adherent and had low CD4 T cell count also suffered from different opportunistic infections. This suggests that individuals may be taking HIV test only after suffering from prolonged illness which could have resulted in a delay in the start of ART regimen. As per the guideline, the patients with low CD4 T cell count should have started their treatment at much earlier date. Efforts are required to ensure that individuals are presented to ART centers without delay. Information related to facilities where HIV testing is available should be spread in the local language. Also, communication campaigns should be conducted at regular intervals to increase awareness about HIV among general population. Linkages between HIV testing centers and ART centers should be established so that HIV infected patients can be put on monitoring of CD4 T cell count without delay. Individuals with frequent illness and sever weakness should be encouraged to take HIV test. Moreover, stigma and discrimination against people living with HIV is prevalent in India which could also have resulted in the delay in treatment seeking. Therefore, community awareness camps should be organized to reduce stigma and discrimination associated with HIV. Engaging frontline health workers to encourage suspected individuals to take HIV test can be a better strategy. Additionally, future research should examine the reasons behind late initiation of ART in the study area so that more concrete strategy can be formulated.

Post-hoc analysis also suggested that a considerable amount of interaction between CD4 T cell count and adherence status. Among individuals with CD4 T cell count <200 cells/cmm, non-adherence increased the mortality risk by 11 times; whereas for those with cell count 200+ cells/cmm, non-adherence increases the risk of mortality by 3 times. This indicates that the baseline CD4 T cell count can influence the effect of adherence on survival of HIV infected individuals. Therefore, initiating ART for individuals as soon as they become eligible is important for success of ART program. Moreover, efforts are required to ensure that patients with low CD4 T cell count consume pills on a regular basis and are provided with nutritional supplements.

This study also documented a higher number of deaths among males as compared to among females. However, we did not observe any differential in adherence status by gender of patients which corroborates earlier research [14]. Increased mortality among male patients on ART has been reported in other studies also [31]–[33]. Empirical research has attributed the higher mortality among males to higher rate of loss to follow-up or delay in the initiation of ART at more advanced stages of AIDS among males as compared to females [31]. However, such differences between male and female patients were minimal in this study. Around 11% males and 7% females were lost to follow up. Similary, average duration between initiation of ART after last HIV test was111 days for males and 108 days for females. Though the duration of initiation of ART from last HIV test was similar for both gender, we noted that median CD4 T cell count was quite lower among males compared to females at the time ART registration. This could have resulted in a higher mortality among males compared to females in the first six months being enrolled into ART. This calls for gender specific approach for ART services. Males should be made aware on symptoms of HIV infection and places where facility for HIV test is available. Early detection of infection may help males to enroll themselves into ART program. Further, biological sex difference in the pharmacology and drug response to ART among males and females can be another reason for higher deaths among males [31].

The findings of the present study should be examined in light of certain limitations. The first limitation of this study was associated with its study design. We used a retrospective cohort and hence we could not take into account all the potential confounders such as viral load, past history of ART treatment, and side effects during medication that could have influenced the association between adherence status and survival of individuals. Another limitation was associated with the cause of death. We considered all cause mortality in the analysis. There is a possibility that a few of the deaths may have occurred due to causes not related to HIV/AIDS. The third limitation was related to the measurement of adherence status. We used the pill count approach to measure the adherence which could have introduced some amount of bias on the measurement of adherence to ART regimen. There is a chance of both under and over-reporting in pill consumption by the patients. However, due to random nature of error in the measurement, we believe that the overall prevalence of adherence status in the study population will remain same. A similar approach to measure adherence has been used in other research conducted in India and elsewhere [10], [12], [16]. Though there is no “gold standard” approach to measure adherence [34], researchers have demonstrated that pill count approach provides very reliable estimates of adherence [35], [36]. Further, use of mean adherence over the period a patient was on treatment could have introduced some degree of measurement bias. Caution is also urged while generalizing the findings to sub-national and national level.

Despite these study limitations, the study findings have important policy implications for ART programs in India. ART services in India are being provided freely to all HIV infected person. The higher survival rate among ART adherent individuals suggests that country's ART program has been successful in increasing longevity of HIV infected individuals. However, there are concerns as more than two-fifths of HIV infected individuals were non-adherent to ART. Efforts are required at the management as well as at implementation level for constant and regular follow-up with all patients. Further, there is a need to increase the risk perception as well as to the knowledge about the benefits of ART among these individuals which can increase the adherence level. In addition, the program should expand the provision of ART services to more remote areas to provide services to some of the underserved population groups like women. Our study also demonstrated higher non-adherence and mortality among individuals with low CD4 T cell counts. This argues for the need to identify and treat eligible HIV infected individuals at an early stage. In addition, late presentation to ART centers is one of main reason for higher mortality in the first six months. Efforts are required to ensure early diagnosis and enrollment into ART for patients with very low CD4 T cell count. Moreover, sensitization at community level is required to reduce stigma and discrimination towards HIV positive individuals so that patients can access treatment without any hesitation.
